# TR1801‐ADC: a highly potent cMet antibody–drug conjugate with high activity in patient‐derived xenograft models of solid tumors

**DOI:** 10.1002/1878-0261.12600

**Published:** 2019-12-03

**Authors:** Marco Gymnopoulos, Oscar Betancourt, Vincent Blot, Ryo Fujita, Diana Galvan, Vincent Lieuw, Sophie Nguyen, Jeanette Snedden, Christine Stewart, Jose Villicana, Jon Wojciak, Eley Wong, Raul Pardo, Neki Patel, Francois D’Hooge, Balakumar Vijayakrishnan, Conor Barry, John A. Hartley, Philip W. Howard, Roland Newman, Julia Coronella

**Affiliations:** ^1^ Tanabe Research Laboratories U.S.A., Inc. San Diego CA USA; ^2^ Spirogen, a member of the AstraZeneca Group London UK

**Keywords:** antibody, drug conjugate, cMet, gastrointestinal cancer, pyrrolobenzodiazepine, solid tumors

## Abstract

cMet is a well‐characterized oncogene that is the target of many drugs including small molecule and biologic pathway inhibitors, and, more recently, antibody–drug conjugates (ADCs). However, the clinical benefit from cMet‐targeted therapy has been limited. We developed a novel cMet‐targeted ‘third‐generation’ ADC, TR1801‐ADC, that was optimized at different levels including specificity, stability, toxin–linker, conjugation site, and *in vivo* efficacy. Our nonagonistic cMet antibody was site‐specifically conjugated to the pyrrolobenzodiazepine (PBD) toxin–linker tesirine and has picomolar activity in cancer cell lines derived from different solid tumors including lung, colorectal, and gastric cancers. The potency of our cMet ADC is independent of MET gene copy number, and its antitumor activity was high not only in high cMet‐expressing cell lines but also in medium‐to‐low cMet cell lines (40 000–90 000 cMet/cell) in which a cMet ADC with tubulin inhibitor payload was considerably less potent. *In vivo* xenografts with low–medium cMet expression were also very responsive to TR1801‐ADC at a single dose, while a cMet ADC using a tubulin inhibitor showed a substantially reduced efficacy. Furthermore, TR1801‐ADC had excellent efficacy with significant antitumor activity in 90% of tested patient‐derived xenograft models of gastric, colorectal, and head and neck cancers: 7 of 10 gastric models, 4 of 10 colorectal cancer models, and 3 of 10 head and neck cancer models showed complete tumor regression after a single‐dose administration. Altogether, TR1801‐ADC is a new generation cMet ADC with best‐in‐class preclinical efficacy and good tolerability in rats.

## Graphical abstract

TR1801‐ADC is an antibody–drug conjugate with highly potent PBD payload–linker conjugated site‐specifically to our cMet antibody. The ADC is stable in rat circulation with acceptable tolerability. TR1801‐ADC is highly active in MET‐amplified and cMet‐overexpressing cancer cell lines. Patient‐derived xenograft solid tumor models of the stomach, colorectum, and head and neck were highly sensitive to treatment with our anti‐cMet‐ADC.

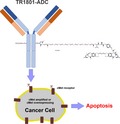

**Abbreviations**
ADC: antibody–drug conjugate
DAR: drug–antibody ratio
ERK: extracellular signal‐regulated kinase‐1
HGF: hepatocyte growth factor
HIC: hydrophobic interaction chromatography
kD: kilo Dalton
MAPK: mitogen‐activated protein kinase
MMAE: monomethyl auristatin E
NAC: N‐acetyl cysteine
PBD: pyrrolobenzodiazepine
PDX: patient‐derived xenograft
PI3K: phosphatidylinositol 3‐kinase
PK: pharmacokinetics
SEC: size‐exclusion chromatography
TFF: tangential flow filtration
TGI: tumor growth inhibition
TMA: tissue microarray
vc: valine–citrulline
VEGF: vascular endothelial growth factor

## 1 Introduction

Antibody–drug conjugates (ADCs) with over 40 years of research are a promising and fast‐growing class of targeted anticancer immunotherapies. These agents combine the specificity of antibodies with the potency of chemotherapeutics by attaching highly cytotoxic payload–linkers covalently to monoclonal antibodies (Mukherjee *et al.*, 2019). With four approved ADCs in the United States for hematological cancers and solid tumors and over 60 ADCs at various stages of development, the field is rapidly expanding and evolving (Beck *et al.*, 2017). The shortcomings of older generation ADCs, mainly serum stability and low tolerability in humans, could be vastly improved by changing and optimizing conjugation chemistry, antibody–drug ratio, and using a new repertoire of linker–toxins (tubulin inhibitors, pyrrolobenzodiazepines (PBDs), irinotecan derivatives, and DNA monoalkylators) (Agatsuma, 2017; Beck *et al.*, 2017; Mantaj *et al.*, 2017; Tolcher, 2016).

The proto‐oncogene MET encodes the receptor tyrosine kinase (cMet). Upon binding of its ligand, hepatocyte growth factor (HGF), a series of intracellular signals are initiated involving morphogenic differentiation, wound healing, motility, invasion, and antiapoptosis (Petrini, 2015; Zhang *et al.*, 2018). Aberrant cMet expression or constitutive activation of the cMet signaling pathway due to amplification, overexpression of its ligand HGF, and mutation in MET is seen in many human tumor types and is the rationale for developing cMet‐targeting therapeutics (Gherardi *et al.*, 2012). Many small molecule cMet inhibitors and pathway‐inhibiting biologics were developed over the last decade with limited or no clinical success (Puccini *et al.*, 2019). The therapeutics that were successful seem to be limited to small subsets of patients with MET‐amplified cancers (Comoglio *et al.*, 2018). Novel cMet‐targeting therapies, such as ADCs, are in development, which are independent of MET amplification status and target any cMet‐overexpressing cancer (Wang *et al.*, 2017; Yang *et al.*, 2019).

TR1801‐ADC is an innovative new generation ADC with highly optimized features, including a DNA‐damaging payload, distinguishing itself from other cMet therapeutics such as ADCs with tubulin inhibitor payload.

## 2 Materials and methods

### 2.1 Cell lines and culture conditions

SNU‐1 (RRID:CVCL_0099), SNU‐16 (CVCL_0076), SNU‐5 (CVCL_0078), NCI‐H1373 (CVCL_1465), NCI‐H1975 (CVCL_1511), NCI‐H1573 (CVCL_1478), NCI‐H441 (CVCL_1561), SW1417 (CVCL_1717), SW‐480 (CVCL_0546), NCI‐H747 (1587), HCT‐116 (CVCL_0291), Detroit 562 (CVCL_1171), and FaDu (CVCL_1218) were purchased from the American Tissue Type Collection (ATCC, Manassas, VA, USA). MKN‐45 (CVCL_0434) was obtained from Deutsche Sammlung von Mikroorganismen and Zellkulturen (DSMZ, Braunschweig, Germany) and SNU‐620 (CVCL_5079) from the Korean Cell Line Bank (KCLB, Seoul, Korea). All cell lines were authenticated at ATCC by analyzing short terminal repeats and found to be correct matches. All experiments were performed with mycoplasma‐free cells. Cell lines were maintained according to the cell bank’s recommendations or in normal growth medium, RPMI‐1640 (Thermo Fisher, #21870092, Waltham, MA, USA) with 2 mm glutamine (Thermo Fisher, #25030164) and 10% FBS (Thermo Fisher, #26140079) at 37 °C and 5% CO_2_.

### 2.2 P3D12 cMet antibody humanization

Five different methods were used to humanize the P3D12 anti‐cMet antibody: CDR grafting, grafting of abbreviated CDRs, SDR transfer, Frankenstein approach, and veneering. The abbreviated CDR method and the SDR method yielded the same amino acid sequence. The resulting four heavy chain and four light chain variable regions were cloned into the pFUSE hIgG2 and pFUSE hκ vectors, respectively, giving 16 possible combinations of heavy/light chain pairs, which were expressed in Expi293 cells. Antibodies were purified, affinities determined, and biophysical characteristics assessed.

### 2.3 Subclass switching and site‐specific cysteine incorporation

The variable regions of the mouse P3D12 antibody were cloned into vectors containing the nucleotide sequence of the constant regions of human IgG1 and human IgG2. The site‐specific cysteines were introduced utilizing nearby restrictions sites and Gibson assembly.

### 2.4 Antibody expression and conjugation

The humanized, IgG2 monoclonal antibody, hD12, was engineered to incorporate an unpaired cysteine residue on each heavy chain Fc region to generate TR1801‐Ab, which was produced from a stably transfected Chinese hamster ovary cell line and purified using protein A affinity chromatography followed by size‐exclusion chromatography (SEC). Prior to conjugation, the hD12 antibody was partially reduced using 495 molar equivalents of L‐glutathione (Sigma‐Aldrich, #G6529, St. Louis, MO, USA), and after incubating for 1 h at room temperature, the glutathione was removed by tangential flow filtration (TFF). For conjugation, 10 molar equivalents of a 10 mm SG3249 DMSO solution were added to the reduced antibody, and the reaction continued for 1 h at room temperature. The reaction was quenched with the addition of a 15‐fold excess of N‐acetyl cysteine (NAC; Acros Organics, #160280250, The Hague, Netherlands) and underwent incubation at room temperature for another 15 min. The unreacted NAC‐capped SG3249 was removed using TFF while buffer exchanging into 25 mm histidine, 85 g·L^−1^ trehalose dihydrate, pH 5.5 buffer. The ADCs, TR1801‐ADC, was recovered and sterile‐filtered using 0.2‐µm PES filter, and polysorbate‐80 (Amresco, # M126, Solon, OH, USA) was added up to a final concentration of 0.02% v/v. The overall product recovery was 87% for the conjugation reaction.

### 2.5 Analytical methods

**Figure 1:**
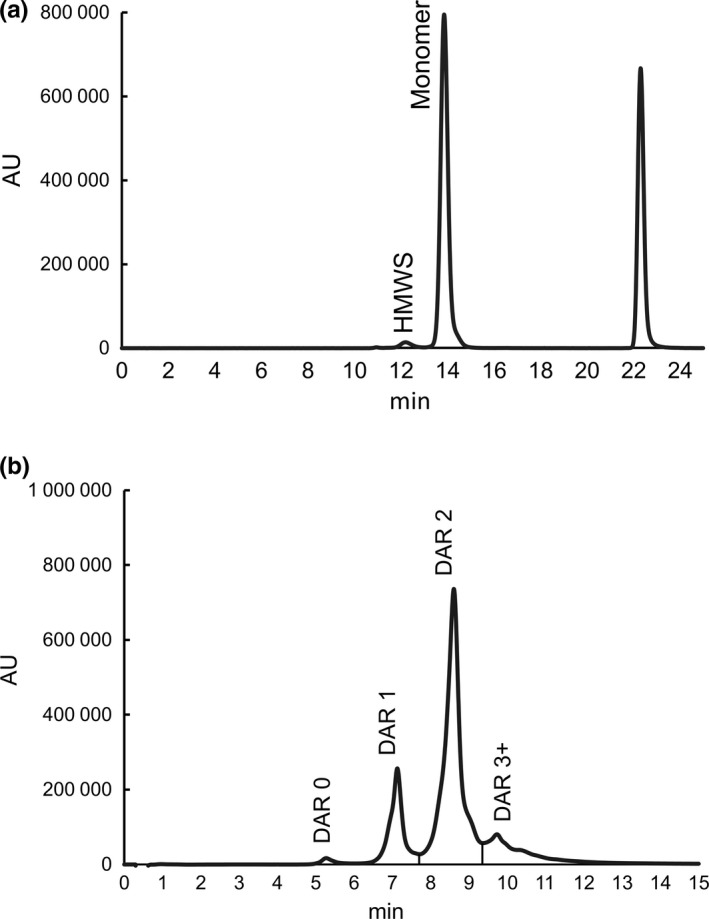
Analytical characterization of TR1801‐ADC. (A) SEC analysis of TR1801‐ADC using a Tosoh Bioscience TSKgel SuperSW mAb column and 200 mm potassium phosphate pH 6.95, 250 mm potassium chloride, and 10% isopropanol (v/v) mobile phase. Note: The peak absorbance at ~ 22.5 min is from the formulation buffer. (B) HIC analysis of TR1801‐ADC using a Butyl‐NP5 column equilibrated with mobile phase buffer A (1.5 m ammonium sulfate, 25 mm sodium phosphate, pH 6.5) and linear elution gradient elution to mobile phase B (25 mm sodium phosphate, pH 6.50, 25% v/v isopropanol).

The concentration of TR1801‐ADC was determined by subtracting the ratio of absorbance values measured at 280 and 330 nm (*A*_280_/*A*_330_) for SG3249 from the *A*_280_ measurement for TR1801‐ADC to account for the absorbance of the conjugated payload. The remainder was divided by the molar extinction coefficient (ε_280_) of the hD12 antibody. To determine the monomer content and weighted average of drug‐to‐antibody ratio (DAR) values, the TR1801‐ADC sample was diluted to 1 mg·mL^−1^ and analyzed using analytical SEC and hydrophobic interaction chromatography (HIC). SEC analysis of TR1801‐ADC using a Tosoh Bioscience TSKgel SuperSW mAb column and mobile phase buffer containing 200 mm potassium phosphate pH 6.95, 250 mm potassium chloride, and 10% isopropanol (v/v) demonstrated ~ 3% high molecular weight species. No low molecular weight species were observed (Fig. 1). The weighted average of DAR value was determined using a HIC Butyl‐NP5 column equilibrated with mobile phase buffer A (1.5 m ammonium sulfate, 25 mm sodium phosphate, pH 6.50). After sample loading and washing, a mobile phase B (25 mm sodium phosphate, pH 6.50, 25% v/v isopropanol) gradient was applied to sequentially elute the low‐DAR to high‐DAR species (Fig. 1). Based on this HIC method, the TR1801‐ADC weighted average of DAR was determined to be 1.96 and < 1 percent of the material was unconjugated (Table S1). Proteolytic digestion (FabRICATOR enzyme; Genovis, Lund, Sweden) of TR1801‐ADC followed by reversed‐phase chromatography (*A*_330_ detection) indicated that > 74% of SG3249 was conjugated to the Fc fragment, and peptide mapping showed the peptide containing the engineered cysteine reside was conjugated to SG2349 and no other SG3249‐conjugated peptides were identified (data not shown).

### 2.6 Met degradation and phospho‐Erk ELISA

Total cMet in SNU‐16 cells was measured with the SECTOR Imager 2400 (MSD, Gaithersburg, MD, USA). Cells were treated with anti‐cMet antibodies and incubated for 24 h. Extracellular signal‐regulated kinase‐1 (ERK) phosphorylation in MKN‐45 cells was measured with the SECTOR Imager 2400. MKN‐45 cells were incubated with cMet antibodies for 15 min. All assays were performed as recommended by the manufacturers.

### 2.7 Cytotoxicity assays

Cell viability was determined by measuring the luminescence after adding the CellTiter‐Glo® 2.0 reagent (Promega, #G9242, Madison, WI, USA). Cancer cells were seeded overnight in growth media and incubated at 37 °C, 5% CO_2_, and 95% humidity. ADCs or the PBD warhead, SG3199, were added in serial dilutions starting with concentrations of 100 nm for ADCs and 10 nm for free drug. Cells were exposed to test articles for 5 days. IC_50_s were calculated by nonlinear regression using sigmoidal curve fitting in prism 7 (GraphPad, San Diego, CA, USA).

### 2.8 *In vivo* tumor xenograft studies in mice

**Figure 2:**
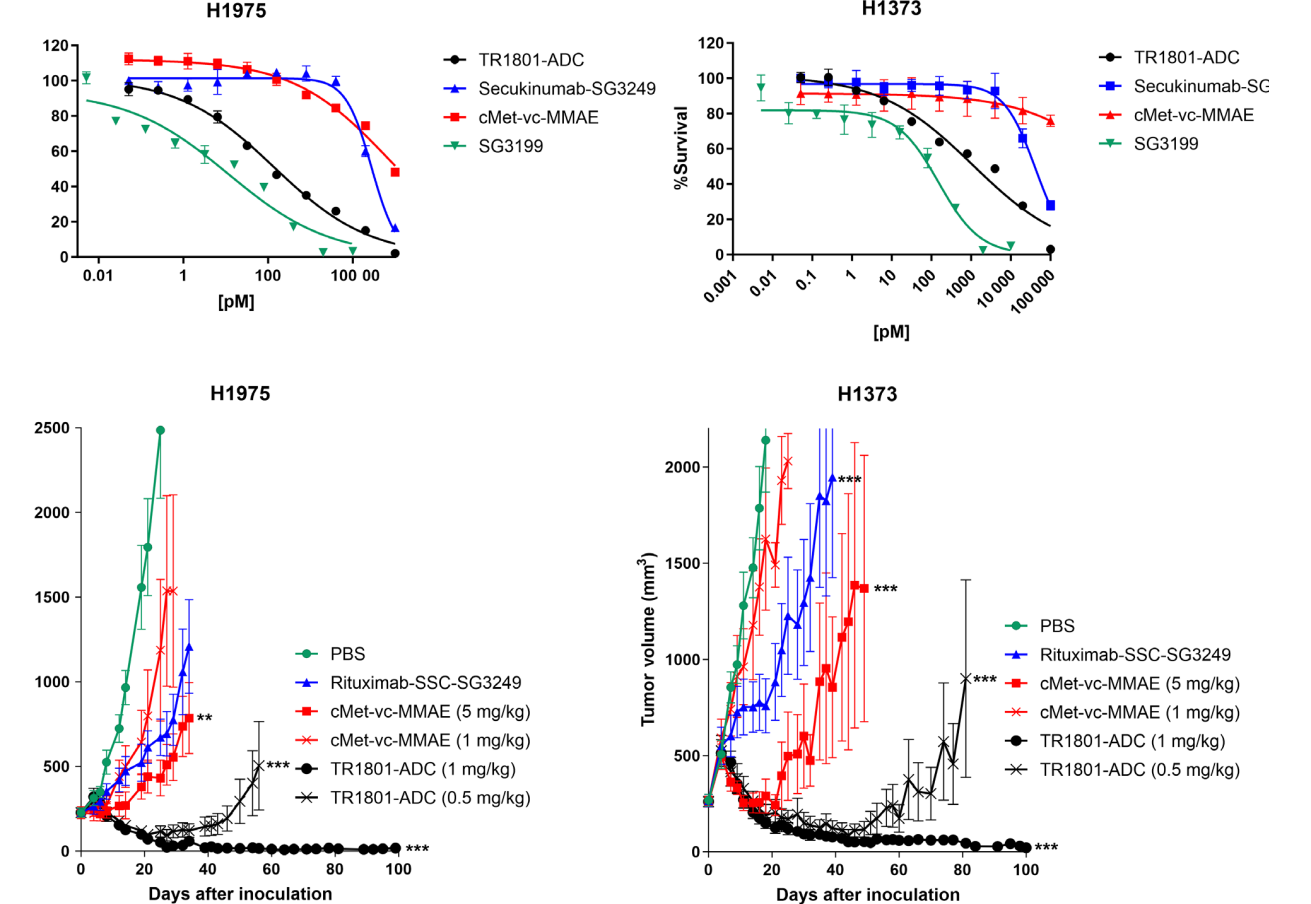
*In vitro* potency and *in vivo* efficacy of TR1801‐ADC with lung cancer cell lines H1975 (60 000 cMet receptors/cell) and H1373 (97 000 cMet receptors/cell) with medium–low cMet expression. Five‐day CellTiter‐Glo® cytotoxicity assays were run as duplicates and repeated at least one time. Lung cancer xenografts in Nu/Nu mice were inoculated with 5 × 10^6^ cells/mouse, and mice were injected with test articles at an average tumor volume of 200–300 mm^3^. Tumor volume is plotted in mm^3^ ± SEM. (A) Lung cancer cell lines H1975 and H1373 were treated with TR1801‐ADC, nontargeting ADC secukinumab–SG3249, cMet‐vc‐MMAE (10‐point dilution series with a starting concentration of 100 nm), or free PBD toxin SG3199 (starting concentration of 10 nm). (B) Lung cancer xenografts H1975 and H1373 were treated with single intravenous doses of vehicle (1× PBS), TR1801‐ADC (1 and 0.5 mg·kg^−1^), cMet‐vc‐MMAE (5 and 1 mg·kg^−1^), and nontargeting ADC (1 mg·kg^−1^) with eight animals per group. Statistics: one‐way ANOVA with Dunnett’s multiple comparison test. Shown is only the significance between cMet‐vc‐MMAE, TR1801‐ADC, and rituximab‐SSC‐SG3249 in comparison with control (*P < 0.05, **P < 0.01, ***P < 0.001).

**Figure 3:**
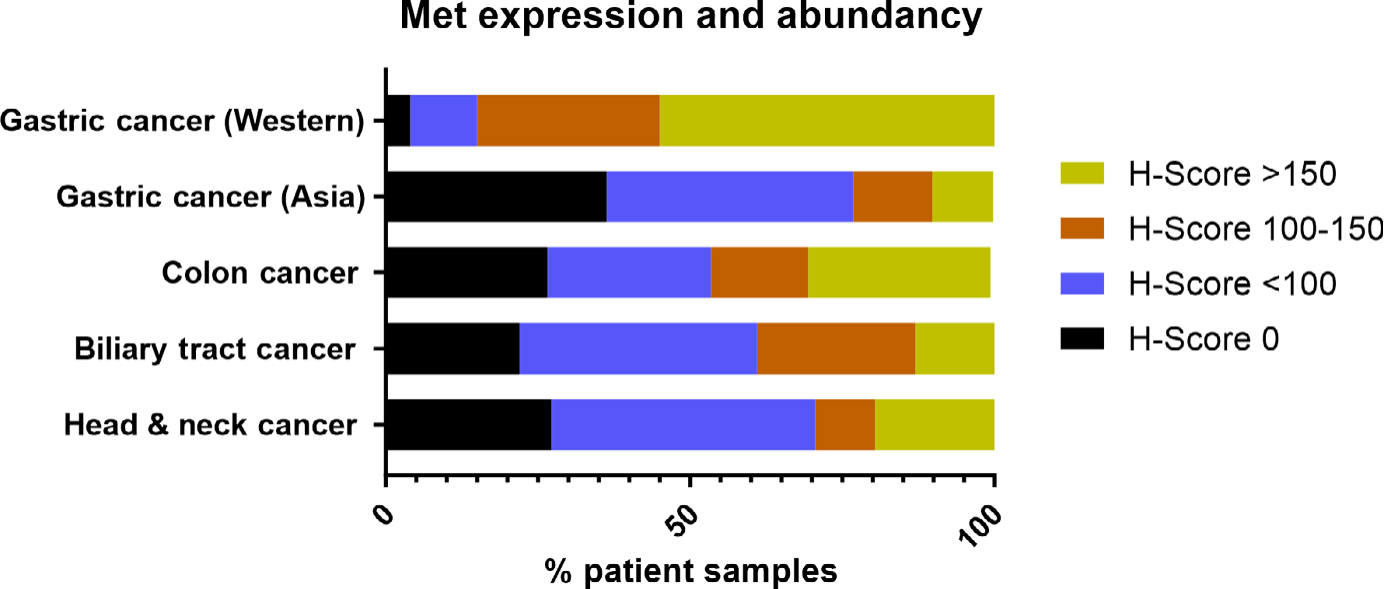
Expression of cMet in patient samples of gastric, colon, biliary tract, and head and neck (H&N) cancers. IHC was performed with SP44 rabbit monoclonal cMet antibody on TMAs (80–100 cores per indication were analyzed). Intensity of cMet staining was scored on a scale from 0 to 300 (*H*‐score) and grouped in four levels of Met expression (none, low, medium, and high) and plotted as % of patient samples.

**Figure 4:**
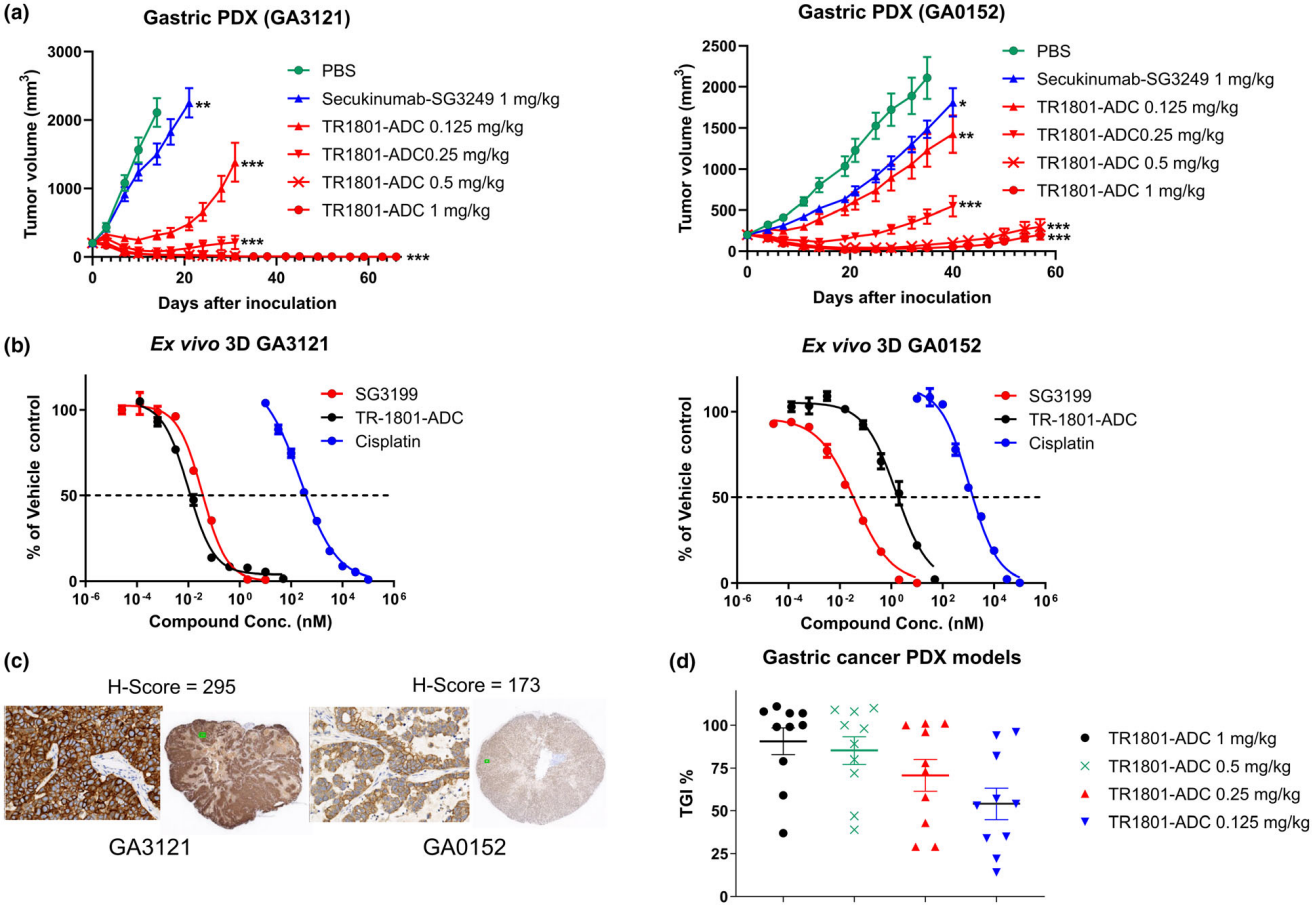
Preclinical assessment of TR1801‐ADC in 10 HuPrime® gastric cancer PDX models. Female BALB/c nude mice were treated with a single intravenous dose of vehicle control, TR1801‐ADC, or nontargeting ADC (secukinumab–SG3249) when subcutaneous tumors reached an average size of 200 mm^3^. *Ex vivo* 3D methylcellulose assays were performed on selected gastric PDX over a 7‐day period with TR1801‐ADC, free PBD toxin SG3199, and cisplatin. Nine‐point dilution series were prepared with starting concentrations of 50, 10, and 100 µm, respectively. Assay was run in triplicates with an *n* = 1. (A) Two representative gastric PDX models GA3121 and GA0152. Tumor growth of each group (*n* = 10) was monitored after a single intravenous administration of vehicle (1× PBS), TR1801‐ADC (1, 0.5, 0.25, and 0.125 mg·kg^−1^), or nontargeting ADC (1 mg·kg^−1^). Statistics: one‐way ANOVA with Dunnett’s multiple comparison test (*P < 0.05, **P < 0.01, ***P < 0.001). (B) *Ex vivo* 3D assay performed with GA3121 and GA0152 PDX models and treated with free PBD toxin (SG3199), TR1801‐ADC, or cisplatin. (C) Representative IHC staining with rabbit monoclonal cMet antibody (SP44) on tissue sections of gastric cancer PDX models GA3121 and GA0152. (D) Plot of 10 gastric cancer PDX models. Tumor growth inhibition (%) at different dose concentrations (1, 0.5, 0.25, and 0.125 mg·kg^−1^) of TR1801‐ADC.

All *in vivo* xenograft studies were approved by the IACUC of Tanabe Research Laboratories, USA, Inc. (San Diego, CA, USA) and performed according to the company’s Institutional Animal Care Guidelines. H1975 and H1373 cancer cell lines were implanted subcutaneously at 5 × 10^6^ cells/animal into the right flank of female Nu/Nu mice obtained from Charles River (Wilmington, MA, USA). Animals were randomized after the average tumor volume reached 200–300 mm^3^. Mice were given a single intravenous injection of ADC, nontargeting control ADC, or vehicle control at doses described in the Figs 1, 2, 3, 4. Body weight and tumor volume were measured 2–3 times per week over the entire duration of the studies. Tumor volume was calculated as follows: V(mm^3^) = 0.5236 × length (mm) × width^2^ (mm). Tumor volumes ± SEM were plotted in prism 7 (GraphPad). Statistical significance was determined with a one‐way ANOVA with Tukey’s or Dunnett’s multiple comparison test dependent on whether groups were compared to a control group or not. When only two dose groups were compared, an unpaired two‐tailed *t*‐test was performed in prism 7.

### 2.9 Rat pharmacology study

Male Sprague Dawley rats were given an intravenous bolus injection of 0.5, 1, 1.5, and 2 mg·kg^−1^ of ADCs. Body weights and general clinical observations were recorded daily over the entire 21 days of the study. Blood samples for pharmacokinetics (PK) were drawn predose, 4, 24, 48, 96, 168, 336, and 504 h after injection of test articles and collected in heparin‐coated tubes, followed by 14 000 ***g*** centrifugation for 5 min. Plasma concentrations of ADCs were measured by ELISA. Noncompartmental pharmacokinetic parameters were calculated using WinNonlin software (Pharsight, Mountain View, CA, USA).

### 2.10 TR1801‐ADC Intact ELISA (ADC) and Total Antibody ELISA (TAB)

For the ADC ELISA, a high‐affinity, anti‐payload‐specific monoclonal antibody was used to capture TR1801‐ADC in serum samples and an anti‐human FC‐specific secondary antibody was used for detection. This assay format allowed for the detection of TR1801‐ADC species containing either 1 or 2 conjugated payloads but did not detect unconjugated TR1801‐ADC species. For the TAB ELISA, a purified extracellular domain of cMet was used as the coating reagent to capture TR1801‐ADC in serum samples *via* antibody–antigen interactions. The immobilized TR1801‐ADC species were detected using an anti‐human secondary antibody specific for kappa light chains. Since site‐specific conjugation of the payload to the attachment site on the heavy chain did not affect antigen binding, this assay format demonstrated equivalent detection of both payload‐conjugated and unconjugated TR1801‐ADC species. For the TAB ELISA, the lower limit of quantification (LLOQ) in rat serum is 15 ng·mL^−1^ and the minimum required dilution (MRD) is 10‐fold, while for the ADC ELISA, the LLOQ is 25 ng·mL^−1^ and the MRD is 100‐fold. Comparison of the individual TR1801‐ADC serum concentrations and/or relevant toxicokinetic parameters (e.g., *C*_max_ and AUC) determined using the ADC and TAB ELISAs could provide insights into potential deconjugation of the payload after dosing. The TAB‐to‐ADC ratio of ~ 1 would indicate unconjugated TR1801‐ADC species were not detected, and the observed TR1801‐ADC toxicokinetic properties were consistent between assays. A TAB‐to‐ADC ratio demonstrably > 1 would suggest the presence of unconjugated TR1801‐ADC species and potential loss of the payload.

### 2.11 IHC of tissue microarrays (TMAs) and PDX sections

TMAs (US Biomax, Derwood, MD, USA, or US Biolabs, Rockville, MD, USA) or patient‐derived xenograft (PDX) cancer tissue sections (CrownBio, Beijing, China) were treated at 100 °C in EDTA buffer pH 9 for 20 min for antigen unmasking. Primary rabbit monoclonal antibody cMet SP44 (Abcam, #ab227637, Cambridge, MA, USA) (diluted 1 : 200) or rabbit IgG isotype control (1 : 200) (Abcam, #ab27478) were incubated with tissue sections for 60 min at room temperature. Goat anti‐rabbit IgG‐ horseradish peroxidase conjugate was used as a detection antibody (Leica Biosystems, #DS9800, Wetzlar, Germany) at 25 µg·mL^−1^ concentration for 60 min at room temperature. All stained TMAs were scanned with the NanoZoomer Image system® (Hamamatsu, Hamamatsu City, Japan), and IHC staining intensity was scored according to following formula: Total score = (% at 0) × 0 + (% at 1) × 1 + (% at 2) × 2 + (% at 3) × 3 with 0 = no staining, 1 = weak staining, 2 = medium staining, and 3 = strong staining.

### 2.12 PDX cancer models

cMet ADCs were evaluated in HuPrime® cancer PDX models (CrownBio) in female BALB/c nude mice (14–15 weeks old). Each mouse was subcutaneously inoculated at the right flank with a 2–3 mm (diameter) tumor piece of one of the tested PDX models. Mice were randomly grouped into six groups (*n* = 10 animals) according to the tumor size average of 200 mm^3^. A single dose (otherwise it is indicated when more than one dose was given) of test articles was administered intravenously into the tail vein at the dose concentrations indicated. Animals were checked daily for morbidity and mortality. Tumor size was measured twice a week with calipers. Tumor volume was calculated using the formula TV = 0.5 × *A* × *B* ^2^. prism 7 (GraphPad) was used to perform a one‐way ANOVA and Dunnett’s multiple comparison analysis to calculate *P* values. TGI% was calculated as follows: TGI% = (mean (control day ×)‐mean (control day 0))‐(mean (test article day ×)‐mean (test article day 0)/ (mean (control day ×)‐ mean (control day 0)*100.

### 2.13 PDX 3D *ex vivo* experiments

Single‐cell suspensions were isolated from HuPrime® cancer PDX models (CrownBio) from tumors that reached a volume of 500–800 mm^3^. 2 × 10^5^ cells were mixed with 1% methylcellulose and seeded into a 96‐well plate. Plates were incubated overnight at 37 °C with 5% CO_2_ and 95% humidity. Test articles were added and incubated for 7 days. Cell viability was determined by adding CellTiter‐Glo® reagent (Promega, #G7572) and reading luminescence on an EnVision Multilabel Reader (PerkinElmer, Waltham, MA, USA). Data were displayed in prism 7 (GraphPad), and IC_50_s were calculated by using a nonlinear regression model with sigmoidal fitting.

## 3 Results

### 3.1 Humanization of P3D12 antibody and subclass switching to reduce agonist activity and potential immunogenicity

The mouse, anti‐human cMet antibody P3D12 was selected as the lead antibody because of its high‐affinity binding to humans and cynomolgus monkey (0.8 nm kD), and rat cMet (15.6 nm kD) (Table S2), which allowed to investigate the tolerability of ADCs in another species besides nonhuman primates. By switching the subclass from IgG1 to IgG2, the agonist activity was significantly reduced (~ 50%) to minimize mitogen‐activated protein kinase (MAPK) and phosphatidylinositol 3‐kinase (PI3K) pathway activation (Fig. S1). The humanization of P3D12 (hD12) had no negative impact on affinity (0.26 nm kD for human cMet, 7 nm kD for rat cMet), internalization, or nonagonist activity compared to the parental mouse antibody (Fig. S1, Table S2).

### 3.2 Five cleavable PBD linker–toxins stochastically conjugated to wt hD12 showed different activities *in vitro* and *in vivo*

To choose the optimal linker–toxin for our cMet ADC, TR1801‐ADC, with respect to activity and tolerability, five cleavable (Val‐Ala) PBD linkers from Spirogen Ltd (London, UK). were conjugated and evaluated using the wt hD12 cMet antibody (rat and cynomolgus monkey cross‐reactive) for their activity *in vitro* and *in vivo,* and tolerability in rats (Fig. S2). hD12‐SG3259 exhibited the highest activity (IC_50_ = 24.4 pm and 86% maximal killing) in the H1975 (60 000 cMet/cell) lung cancer cell line followed by hD12‐SG3246 (IC_50_ = 91 pm, 62%), hD12‐SG3315 (IC_50_ = 146 pm, 46%), and hD12‐SG3249 (IC_50_ = 153 pm, 79%; Table S3, Fig. S2A). The hD12‐SG3227 ADC was the least active (IC_50_ = 410 pm, 77%). The difference in potencies was less prominent in another cMet‐expressing lung cancer cell line H1373 (97 000 cMet/cell) with IC_50_ of 11 pm (SG3246), 20 pm (SG3315), 22 pm (SG3249), 28 pm (SG3227), and 31 pm (SG3259) (Fig. S2A, Table S3). The differences in potency seen *in vitro* with H1975 cells matched the efficacy seen in the H1975 subcutaneous mouse xenograft model (Fig. S2B). All ADC variants had significant antitumor activity compared to PBS control (*P* < 0.0001) in all dose groups (Table S4). While single intravenous high doses (0.5 mg·kg^−1^) of cMet ADC variants lead to full tumor regression in all groups with no significant differences, the lower dose groups (0.125 mg·kg^−1^) differed in antitumor activity. hD12‐SG3259 was significantly more potent at the low dose than SG3315 (*P* < 0.0001) and SG3227 (*P* < 0.0001); but not in comparison with SG3249 and SG3246 (Table S4). In general, hD12‐SG3259, hD12‐SG3249, and hD12‐SG3246 were the most potent, while hD12‐SG3227 and hD12‐SG3315 were the least potent. Based on these data, SG3249 and SG3259 were chosen as the lead PBD toxin–linker candidates and further investigated in a rat tolerability study. SG3259‐conjugated cMet ADC (hD12‐SG3259) was less well tolerated than hD12‐SG3249. Animals were found moribund or dead after 7 or 10 days in the high‐dose groups of hD12‐SG3259, and rapid body weight loss over 20% was observed after 7 days. hD12‐SG3249 ADC was well tolerated in all dose group with no significant body weight loss or any other clinical observations (Fig. S2C). Thus, SG3249 (tesirine) was selected as lead PBD toxin–linker for all further studies.

### 3.3 Site‐specific conjugation of SG3249 to hD12 produced a stable and homogeneous ADC

In general, site‐specific conjugates are more homogenous drugs and have several benefits over stochastically linked toxin–linkers. The stability of site‐specific ADCs is highly increased, which often leads to improved tolerability and a better PK profile *in vivo* (Strop *et al.*, 2015; Strop *et al.*, 2013). The heavy chain constant region of hD12 was used, and tesirine was conjugated site‐specifically to introduced cysteines in the CH2 domain. The product was a homogeneous ADC (97% monomeric) with a DAR of 2 (average DAR 1.96) (Fig. 1, Table S1). Site‐specific hD12 SG3249 conjugate was slightly more potent *in vitro* than stochastically conjugated hD12‐SG3249 (32 versus 153 pM) (Table S3). Both ADCs showed similar and significant *in vivo* antitumor activity at both dose levels compared to a control (*P* < 0.0001) (Fig. S3A and S3B). The circulating terminal half‐life for TR1801‐ADC was 14 days in rats (Fig. S3C). Comparing the serum concentrations measured using a Total Antibody PK Assay, which detects both conjugated and unconjugated antibody species, and an ADC PK Assay, which detects only conjugated species, significant deconjugation of payload–linker was not observed. The tolerability in rats was good with continuous body weight gain in all dose groups and no remarkable clinical observations (Fig. S3D). The site‐specific cMet hD12–tesirine conjugate was named TR1801‐ADC.

### 3.4 TR1801‐ADC was potent in 14 cMet‐expressing cancer cell lines and two xenografts with medium–low cMet expression

Fifteen cancer cell lines from different organs (gastric, colorectal, and head and neck and lung cancers) were tested for sensitivity to TR1801‐ADC. The cMet expression levels ranged from zero (SNU‐1), low (SNU‐16), medium (H1373) to high (MKN‐45). IC_50_s varied between 4 pm (H441) and 13 nm (H1573) (Table 1).

**Table 1:** Potency and efficacy of TR1801‐ADC in 15 cancer cell line with various cMet expression levels and MET amplification status. Cancer cell lines were exposed to TR1801‐ADC for 5 days before CellTiter‐Glo® reagent was added. IC_50_s and % maximum killing were determined in graphpad prism 7 after sigmoidal curve fitting of dose–response curves.

Cell line	Cancer type	MET copy number (CCLE)	MET receptor #	TR1801‐ADC IC50 (pm)	SD	% max kill	SD	*n*
SNU‐5	Gastric	3	460 000	15.6	2.7	97.5	0.4	2
MKN‐45	Gastric	12	295 000	15.8	6.7	97.8	1.6	4
SNU‐620	Gastric	45	294 000	197.9	67.3	99.3	0.3	3
H1373	Lung	3	97 000	2272.7	857.0	95.5	1.2	6
H441	Lung	3	74 000	4.2	1.4	95.2	3.1	2
H1573	Lung	21	73 000	13 444.8	20 146.4	88.8	2.3	3
H1975	Lung	3	60 000	346.4	192.1	97.6	1.7	5
Detroit 562	Head and neck	4	59 000	11 030.5	7547.0	68.3	2.5	2
H747	Colorectal	2	52 000	3230.0	192.3	99.1	0.1	2
SW1417	Colorectal	3	38 000	3494.0	1887.9	92.9	2.7	4
SNU‐16	Gastric	3	37 000	4664.0	478.0	90.5	6.2	2
HCT116	Colorectal	2	37 000	190.2	58.1	98.9	0.9	3
FaDu	Head and neck	2	34 000	327.5	4.7	98.1	0.0	2
SW480	Colorectal	2	5000	1380.0	90.5	92.4	10.3	2
SNU‐1	Gastric	2	0	24 373.7	3677.2	97.4	2.0	3

Two lung cancer cell lines H1975 and H1373 with medium–low cMet expression and no MET gene amplification (60 000 cMet/cell and 97 000 cMet/cell) are sensitive to TR1801‐ADC, with IC_50_s of 320 pm (98% maximal killing), with H1975 of 2.2 nm (96%), and with H1373 cancer cell lines in cytotoxicity assays (Table S3). A cMet ADC, based on stochastically conjugated cleavable monomethyl auristatin E [cMet‐valine–citrulline (vc)‐MMAE], showed low activity (IC_50_ = 68 nm and > 100 nm with 52% and 29% maximal killing in H1975 and H1373). This was comparable to nontargeting secukinumab–SG3249 (IC_50_ = 26 nm and 48 nm and 83% or 68% maximal killing; Fig. 2A, Table S3). This difference in activity was also seen in the corresponding xenografts (Fig. 2B) and was significant using an unpaired two‐tailed t‐test comparing dose groups of the H1975 study (*P* = 0.0029, high‐dose groups; *P* = 0.023, low‐dose groups) and in the low‐dose group of the H1373 model (*P* < 0.0001; Table S5). Overall, TR1801‐ADC (at 0.5 and 1 mg·kg^−1^) and cMet‐vc‐MMAE (at 5 mg·kg^−1^) showed significant antitumor activity in both models in comparison with the PBS control (Table S5). However, cMet‐vc‐MMAE, even though dosed five times or two times higher (5 and 1 mg·kg^−1^) than TR1801‐ADC, did not cause full tumor regression in either model. TR1801‐ADC showed complete tumor regression in the highest dose (1 mg·kg^−1^) and partial tumor regression in the low dose (0.5 mg·kg^−1^). The response was not only more pronounced with TR1801‐ADC but also more durable with no tumor regrowth over 100 days in the 1 mg·kg^−1^ group (Fig. 2B).

### 3.5 cMet is highly expressed in cancers of the gastrointestinal tract and head and neck cancers

cMet is highly expressed in several solid tumor types, for example, lung, renal cell, esophageal, and others (Kim *et al.*, 2017; Pyo *et al.*, 2016; Sweeney *et al.*, 2002; Tsao *et al.*, 1998; Xu *et al.*, 2015). We stained TMAs of four different cancer indications of interest (gastric, colon, biliary, and head and neck cancers) and quantified cMet expression. cMet expression was seen in all four indications, but the largest patient sample number with high cMet expression (*H*‐score > 150) was seen in Western population samples of gastric cancer (55%), colon cancer (30%), and head & neck (20%) and biliary cancers (13%) (Fig. 3). Based on these results, gastric, colorectal, and head and neck cancers were selected for translational PDX studies.

### 3.6 GI cancer PDX models are highly sensitive to TR1801‐ADC *in vivo* and *ex vivo*

To further substantiate the antitumor activity of TR1801‐ADC in more translatable *in vivo* models, 10 gastric cancer PDX models were chosen with different expression levels of cMet. Two representative models GA3121 and GA0152 are shown with high (*H*‐score 295) and medium–high (*H*‐score 173) cMet expression (Fig. 4C). A durable complete response was seen in both subcutaneous models at 1 and 0.5 mg·kg^−1^ using a single intravenous dose of TR1801‐ADC. Partial responses were seen with single low doses of TR1801‐ADC at 0.25 and 0.125 mg·kg^−1^ (Fig. 4A). In comparison, nontargeting secukinumab–SG3249 had only a minor effect on tumor growth. The *in vivo* response to TR1801‐ADC correlated well with the response *ex vivo*. TR1801‐ADC was slightly less potent in the GA0152 PDX model (medium–high cMet) *in vivo* and had a higher IC_50_ *ex vivo* of 1.7 nm (98% maximal killing) in comparison with 12 pm (99%) in the GA3121 model (high cMet; Fig. 4B). The free toxin SG3199 had similar IC_50_s in both PDX models of 29 pm (100%) and 36 pm (99%) for GA3121 and GA0152. The control test article cisplatin was less potent with an IC_50_ in the 3–4 digit nm range (366 nm for GA3121 and 1460 nm for GA0152). Significant tumor growth inhibition (TGI) was seen in all models at 1 and 0.5 mg·kg^−1^ ranging from > 100% to 40% (Fig. 4D, Table S6). Seven of 10 gastric PDX showed complete tumor regression at 1 mg·kg^−1^, and 5/10 models, at 0.5 mg·kg^−1^.

**Figure 5:**
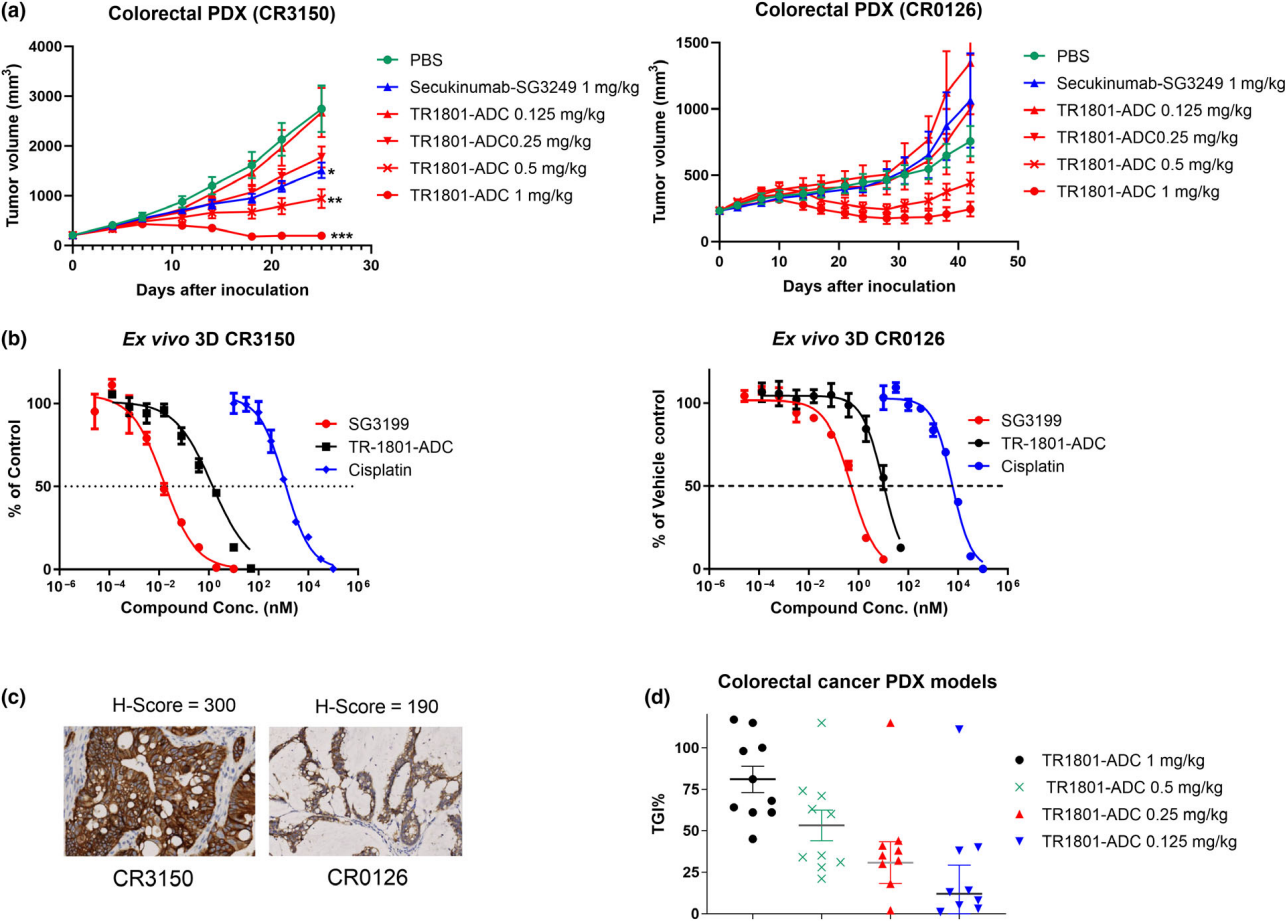
Preclinical assessment of TR1801‐ADC in 10 HuPrime® colorectal cancer PDX models. Female BALB/c nude mice were treated with a single dose of vehicle control, TR1801‐ADC, or nontargeting ADC (secukinumab–SG3249) when subcutaneous tumors reached an average size of 200 mm^3^. *Ex vivo* 3D methylcellulose assays were performed on selected colorectal PDX over a 7‐day period with TR1801‐ADC, free PBD toxin SG3199, and cisplatin. Nine‐point dilution series were prepared with starting concentrations of 50, 10, and 100 µm, respectively. Assay was run in triplicates with *n* = 1. (A) Two representative colorectal PDX models CR3150 and CR0126. Tumor growth of each group (*n* = 10) was monitored after a single intravenous administration of vehicle (1× PBS), TR1801‐ADC (1, 0.5, 0.25, and 0.125 mg·kg^−1^), or nontargeting ADC (1 mg·kg^−1^). Statistics: one‐way ANOVA with Dunnett’s multiple comparison test (*P < 0.05, **P < 0.01, ***P < 0.001). (B) *Ex vivo* 3D assay performed with CR3150 and CR0126 PDX models and treated with free PBD toxin (SG3199), TR1801‐ADC, or cisplatin. (C) Representative IHC staining with rabbit monoclonal cMet antibody (SP44) on tissue sections of colorectal cancer PDX models CR3150 and CR0126. (D) Plot of 10 colorectal cancer PDX models. TGI% at different dose concentrations (1, 0.5, 0.25, and 0.125 mg·kg^−1^) of TR1801‐ADC.

We also wanted to test whether the high expression of cMet in colon cancer translates into a high antitumor activity of our cMet ADC in colorectal PDX models. As anticipated, TR1801‐ADC was highly active in colorectal cancer PDX models with statistically significant growth inhibition in 9/10 PDX models (Table S6). Complete tumor regression was observed in 40% (4/10) when treated with 1 mg·kg^−1^ of ADC. The other 5/10 colorectal PDX models showed partial tumor regression, and one model showed no significant antitumor response (Fig. 5D, Table S6). The PDX model CR3150 with high cMet expression (*H*‐score = 300) was more responsive to a single intravenous dose of TR1801‐ADC than the model CR0126 with medium–high more heterogeneous cMet expression (*H*‐score = 190) (Fig. 5A,C). The nontargeting control secukinumab–SG3249 had some effect in model CR3150 on tumor growth but on the same level as the four times lower dose of TR1801‐ADC. The corresponding *ex vivo* experiments showed that the free toxin SG3199 had a 26‐fold lower potency in model CR0126 (IC_50_ = 500 pm, 100% maximal killing) in comparison with CR3150 (IC50 = 18 pm, 94%) and TR1801‐ADC was 11 times less potent in CR0126 (IC_50_ = 10.54 nm, 87%) than model CR3150 (IC_50_ = 0.97 nm, 99%) (Fig. 5B). The cisplatin control was less potent with IC_50_s > 1000 nm in both models.

Altogether, TR1801‐ADC was highly active in gastrointestinal cancers with a stable and robust antitumor response.

### 3.7 TR1801‐ADC was active in head and neck PDX models with medium‐to‐high cMet expression

**Figure 6:**
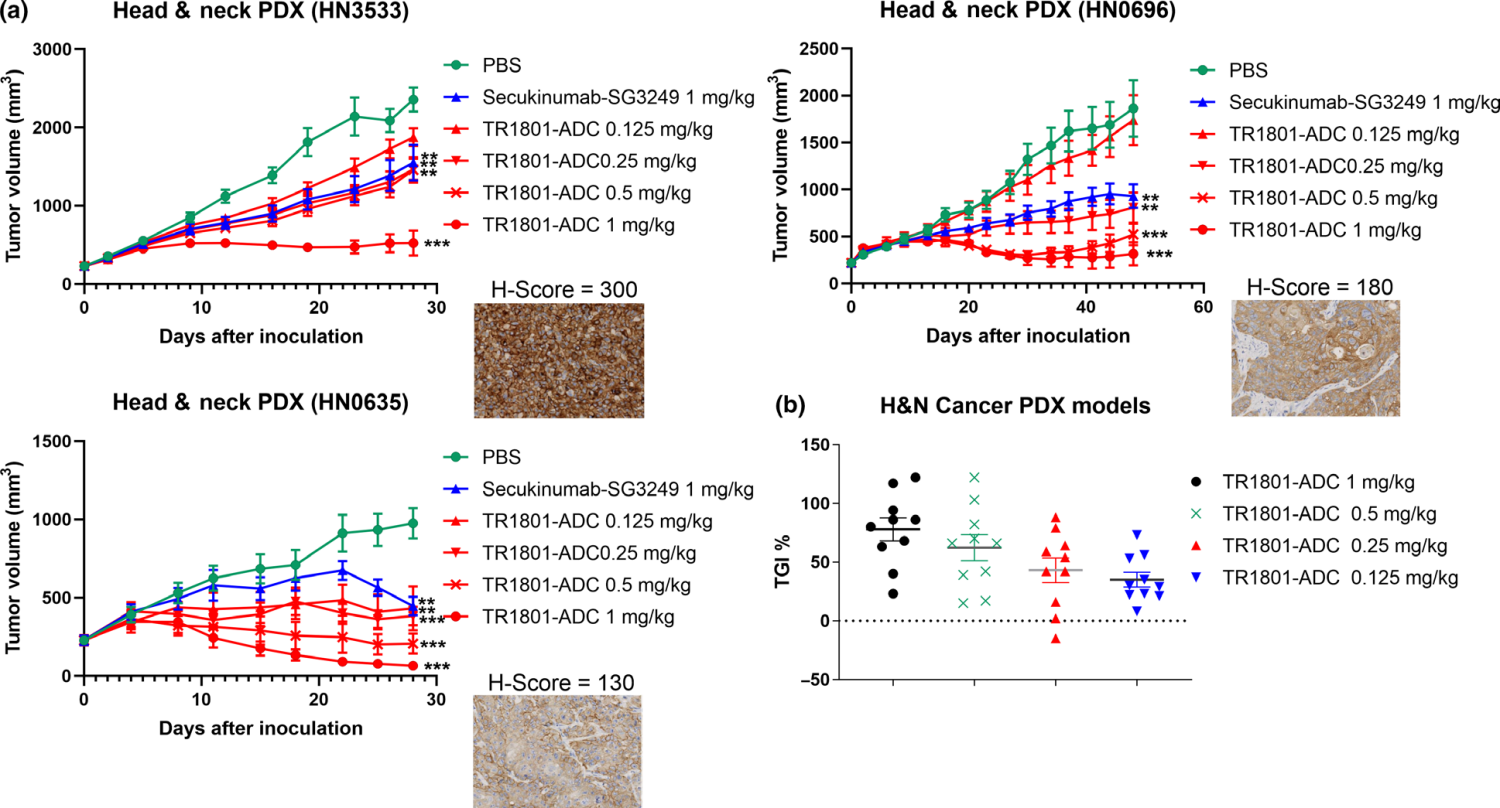
Preclinical assessment of TR1801‐ADC in 10 HuPrime® head and neck cancer PDX models. Female BALB/c nude mice (*n* = 10 per group) were treated with vehicle control, TR1801‐ADC, or nontargeting ADC (secukinumab–SG3249) when subcutaneous tumors reached an average size of 200 mm^3^. (A) Three representative head and neck PDX models HN3533 (*H*‐score = 300), HN0696 (*H*‐score = 180), and HN0635 (*H*‐score = 130) and corresponding IHC with rabbit monoclonal cMet antibody (SP44). Tumor growth of each group (*n* = 10) was monitored after a single intravenous administration of vehicle (1× PBS), TR1801‐ADC (1, 0.5, 0.25, and 0.125 mg·kg^−1^), or nontargeting ADC (1 mg·kg^−1^). Statistics: one‐way ANOVA with Dunnett’s multiple comparison test (*P < 0.05, **P < 0.01, ***P < 0.001). (B) Plot of 10 head and neck cancer PDX models. TGI% at different dose concentrations (1, 0.5, 0.25, and 0.125 mg·kg^−1^) of TR1801‐ADC.

Eight of 10 head and neck PDX models with various levels of cMet expression showed significant growth inhibition in subcutaneous xenografts (Table S6). Complete tumor regression was observed in 30% (3/10) of models when treated with a single dose of 1 mg·kg^−1^ TR1801‐ADC. 50% (5/10) of the models showed partial regression, and two models showed no significant antitumor activity (Fig. 6B, Table S6). The three models with complete tumor regression had cMet expression ranging from high (HN3533 *H*‐score = 300), medium–high (HN0696 *H*‐score = 180), to medium (HN0635 *H*‐score = 130; Fig. 6A). Model HN0635 with medium cMet responded best to TR1801‐ADC treatment.

## 4 Discussion

The HGF receptor, cMet, is highly expressed in various solid tumor indications (Giordano *et al.*, 1992; Isaksson‐Mettavainio *et al.*, 2008; Sierra and Tsao, 2011; Yap *et al.*, 2011), and mutations in the MET gene (Pilotto *et al.*, 2017) (e.g., exon 14 splice variant: NSCLC 3–10%, gastric 7.1%) and gene amplifications are relatively rare (Jardim *et al.*, 2015) (6% gastric cancer, 1% lung cancer). Low normal tissue expression of cMet is seen in the GI tract, liver, skin, and lung, and can increase upon tissue repair and regeneration (Jung *et al.*, 2012; Prat *et al.*, 1991).

cMet expression in tumors is associated with poor prognosis and resistance to targeted therapy, for example, epidermal growth factor receptor and vascular endothelial growth factor pathway inhibitors (Bean *et al.*, 2007; Zhang *et al.*, 2003). There has been a major effort to develop small molecule cMet pathway inhibitors (crizotinib, cabozantinib, capmatinib, tepotinib, and glesatinib) and inhibitory antibodies (onartuzumab, ficlatuzumab, emibetuzumab, SAIT‐301, and ABT700) (Lee *et al.*, 2018a; Puccini *et al.*, 2019). Targeting cMet with small molecule inhibitors and nonagonistic antibodies has not been very successful in the clinic. These therapeutics are limited to MET‐amplified or, more precisely, subsets of cancers with constitutively activated Met pathway. The omission or difficulty of patient stratification contributed to many failed clinical trials (Hughes and Siemann, 2018, 2019). These setbacks opened up opportunities for other targeted therapies that are independent of MET amplification and Met pathway activity such as ADCs (Wang *et al.*, 2017; Yang *et al.*, 2019). The antibody used in TR1801‐ADC is a nonagonistic binder that enhances the safety of our ADC by reducing downstream signaling of cMet (PI3K/AKT, ERK/MAPK and SRC/focal adhesion kinase pathways) and potential activation of tumor‐promoting events (Greenall *et al.*, 2012; Organ and Tsao, 2011). We decided to use the PBD payload tesirine (SG3249) on TR1801‐ADC, which showed the best compromise between potency and tolerability in our studies. Tesirine is currently in preclinical development for several solid tumors and hematological cancers (Cho *et al.*, 2018; Hartley *et al.*, 2018; Tiberghien *et al.*, 2016) and in clinical trials (Horwitz *et al.*, 2017; Rudin *et al.*, 2017). Tesirine is a newer generation PBD (Tiberghien *et al.*, 2016) with reduced hydrophobicity and same potency as talirine (SGD‐1910). Increasing hydrophilicity of the payload leads potentially to a better tolerated toxin by reducing off‐target toxicity (Lucas *et al.*, 2018; Nakada *et al.*, 2019). The site‐specific conjugation of SG3249 to engineered cysteines on the constant region of the heavy chain led to a homogeneous ADC with high stability and long half‐life in rats. It was well tolerated in rats with no major clinical findings or pronounced weight loss even though cMet might be expressed in some normal tissues of the rat (liver, lung, GI tract). More extensive pharmacological studies, especially in nonhuman primates, will have to clarify to which extent normal cMet expression will contribute to on‐target toxicity. Based on preclinical and clinical studies with tesirine as payload (Cho *et al.*, 2018; Rudin *et al.*, 2017; Udagawa *et al.*, 2019), off‐target toxicity will most likely be the critical factor that will determine the maximum tolerated dose in animals and humans.

TR1801‐ADC was potent and highly efficacious in *in vitro and in vivo* experiments with medium–low cMet expression cancer cell lines (H1975 and H1373) in which a cMet ADC with MMAE payload was less active even at higher dose concentrations. We theorize that the receptor number was the driver of activity in this case and not the sensitivity of the cancer to the mechanism of action. This demonstrates that PBD payloads clearly outperform less potent tubulin inhibitor payloads in medium–low‐expressing tumor models. Any concern that the higher potency could cause a lower tolerability in animals was unsubstantiated as TR1801‐ADC, which is cross‐reactive to rat cMet, was well tolerated in a rat pharmacology study.

Taxanes in general and ADCs with tubulin inhibitor payloads (e.g., trastuzumab‐DM1) had low activity in tumors of the gastrointestinal tract as shown in several clinical trials and meta‐analysis (McClelland *et al.*, 2009; Quiles *et al.*, 2010; Shi *et al.*, 2017; Swanton *et al.*, 2009; Swanton *et al.*, 2006). We can assume that cMet ADCs with tubulin inhibitor toxins would share the same fate despite the high and abundant cMet expression in GI cancers and TR1801‐ADC takes advantage of this opportunity. We were able to reproduce published data that showed high and abundant expression of cMet in GI cancers (Gayyed *et al.*, 2015; Lee *et al.*, 2018b; Paliga *et al.*, 2017; Safaie Qamsari *et al.*, 2017; Wu *et al.*, 2014; Yildiz *et al.*, 2016) by performing IHC on TMAs. *Ex vivo* experiments revealed a high activity of TR1801‐ADC in gastric and colorectal PDX models. The sensitivity of TR1801‐ADC was dependent on the activity of the PBD warhead (SG3199) in the tested PDX *ex vivo* models. Reasons for the differences in PBD sensitivity in the PDX models we examined are unknown; however, recently published work (Hartley *et al.*, 2018) may indicate important factors. For example, it was shown that certain defects in the DNA repair protein excision repair cross‐complementation group 1 or homologous recombination repair can sensitize cancer cells to the toxin–linker warhead SG3199 of tesirine. In the opposite case, expression of the multidrug resistance gene 1 (P‐gp) could lower the sensitivity to SG3199 (Hartley *et al.*, 2018).

Based on the encouraging *ex vivo* results, we tested the *in vivo* activity of TR1801‐ADC in gastric, colorectal, and head and neck PDX models with cMet expression between *H*‐scores 130 and 300, the assumed range for robust antitumor activity of TR1801‐ADC. Significant antitumor activity was seen in 90% of all PDX models tested, and full tumor regression was seen in the majority of gastric cancer PDX models and a significant number of colorectal and head and neck cancer models. There was no clear dependency between cMet expression and response to TR1801‐ADC other than that some amount of cMet must be expressed to make the ADC efficacious. A thorough analysis of molecular factors in the tested PDX models that could modulate sensitivity as described recently for SG3199 would be of great interest and could assist to stratify future cMet‐positive patients that will benefit the most from TR1801‐ADC (Hartley *et al.*, 2018; Hughes and Siemann, 2019).

## 5 Conclusions

These data show that TR1801‐ADC could become a best in class therapeutic for the treatment of cMet‐overexpressing tumors and can target tumors with even low cMet expression. Currently, TR1801‐ADC is being assessed in a phase 1 clinical trial for cMet‐overexpressing solid tumors.

## Conflict of interest

The authors declare no conflict of interest.

## Author contributions

MG designed the studies. OB, RF, DG, VL, SN, CS, JV, JW, and EW contributed to the execution of the experiments. MG and OB performed the analysis and interpretation of the data. NP, FD, BV, and CB produced the ADC and performed analytical testing. MG and OB drafted the manuscript. VB, NP, JH, PH, RN, and JC reviewed the manuscript.

## Supporting information


**Fig. S1** **.** Nonagonist activity and *in vitro* degradation of humanized IgG2 cMet antibody hD12.
**Fig. S2** **.** PBD toxin linker assessment *in vitro* and *in vivo* on cMet hD12 antibody.
**Fig. S3** **.** *In vitro* and *in vivo* assessment of site‐specific cMet hD12‐SSC‐SG3249 in comparison to stochastic hD12‐SG3249.
**Table S1** **.** TR1801‐ADC quality attributes.
**Table S2** **.** Affinity and species cross‐reactivity of mouse and humanized cMet P3D12 antibody clone.
**Table S3** **.** Cytotoxicity of TR1801‐ADC, cMet ADC variants and control ADCs with H1975 and H1373 cancer cell lines.
**Table S4** **.** Significance of antitumor activity of hD12 PBD drug‐linker variants in a H1975 xenograft model.
**Table S5** **.** Significance of antitumor activity of TR1801‐ADC at different concentrations and stochastically coupled cMet‐vc‐MMAE ADC in H1975 and H1373 xenograft models.
**Table S6** **.** Significance of antitumor activity of TR1801‐ADC in PDX models.
