# Supplementary material for: TR1801‐ADC: a highly potent cMet antibody–drug conjugate with high activity in patient‐derived xenograft models of solid tumors
Source: Mol Oncol. 2019 Dec 3;14(1):54–68. doi: 10.1002/1878-0261.12600 (PMC6944112; doi:10.1002/1878-0261.12600)
Supplement: Supplementary file 1 — Fig. S1 . Nonagonist activity and in vitro degradation of humanized IgG2 cMet antibody hD12. Fig. S2 . PBD toxin linker assessment in vitro and in vivo on cMet hD12 antibody. Fig. S3 . In vitro and in vivo assessment of site‐specific cMet hD12‐SSC‐SG3249 in comparison to stochastic hD12‐SG3249. Table S1 . TR1801‐ADC quality attributes. Table S2 . Affinity and species cross‐reactivity of mouse and humanized cMet P3D12 antibody clone. Table S3 . Cytotoxicity of TR1801‐ADC, cMet ADC variants and control ADCs with H1975 and H1373 cancer cell lines. Table S4 . Significance of antitumor activity of hD12 PBD drug‐linker variants in a H1975 xenograft model. Table S5 . Significance of antitumor activity of TR1801‐ADC at different concentrations and stochastically coupled cMet‐vc‐MMAE ADC in H1975 and H1373 xenograft models. Table S6 . Significance of antitumor activity of TR1801‐ADC in PDX models. [file MOL2-14-54-s001.docx]

**Supplemental Information**

**Supplemental Figures and Tables**

**Suppl. Figure 1.**

**
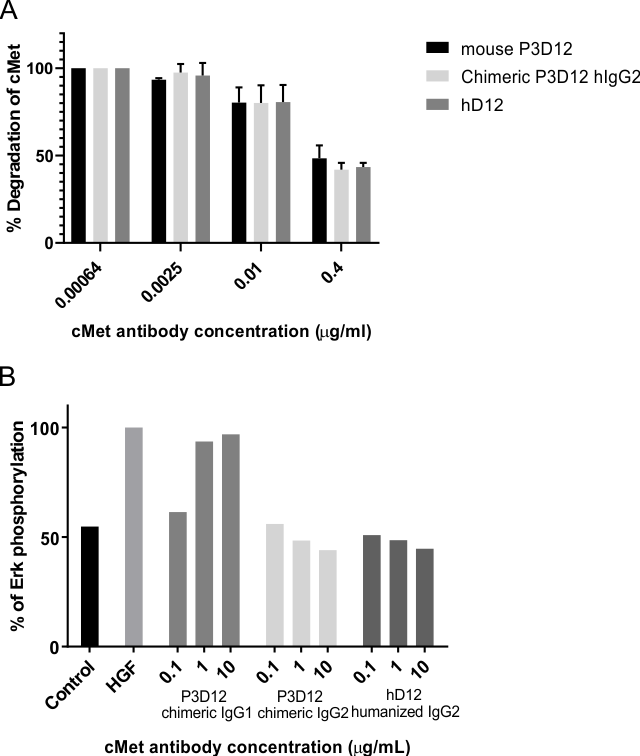
**

**Supplemental Figure S1.**

cMet antibody characteristics were improved or unchanged after humanization and subclass switching. **(A)** Differences in cMet degradation (n=2) through internalization and lysosomal trafficking between mouse cMet antibody P3D12, chimeric hIgG2 P3D12 and humanized P3D12 (hD12). **(B)** Agonistic activity assessed by degree of ERK phosphorylation with chimeric P3D12 (hIgG1), chimeric P3D12 (hIgG2) and humanized P3D12(hD12) (hIgG2).

**Supplemental Figure S2.**


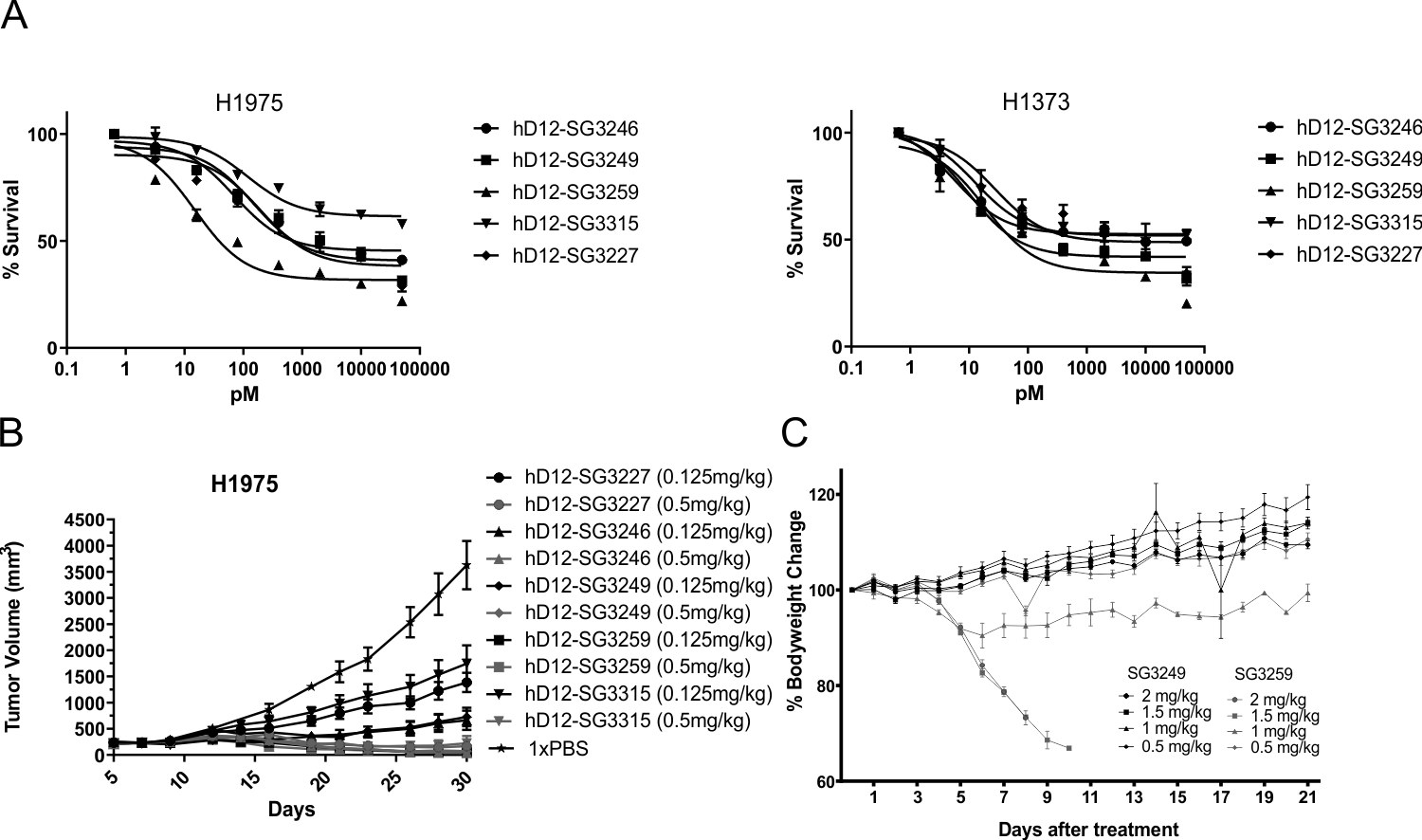


**Suppl. Figure S2.**

PBD toxin linker assessment *in vitro* and *in vivo* on cMet hD12 antibody. 5 cleavable PBD toxin-linkers (SG3249, SG3259, SG3315, SG3246 and SG3227) were stochastically conjugated to hD12 cMet antibody. **(A)** 2 cMet-positive lung cancer cell lines H1975 and H1373 were treated with 7-point dilutions of the 5 cMet ADC variants starting at 50 nM. Viability was determined after 5 days with Cell Titer Glo^®^ in duplicates and repeated at least once. **(B)** Female Nu/Nu nude mice (n=10 per dose group) were injected with test articles (5 cMet PBD toxin variants or vehicle control) after subcutaneous H1975 lung cancer tumors reached an average of 250 mm^3^. **(C)** Male Sprague-Dawley rats (n=3 per dose group) were treated with 2, 1.5, 1 and 0.5 mg/kg of hD12-SG3249 or hD12-SG3259 *via* intravenous bolus injection, and bodyweight and clinical observations were performed daily up to day 21.

**Suppl. Figure S3.**





**Suppl. Figure S3.**

*In vitro* and *in vivo* assessment of site-specific (SSC) cMet hD12-SSC-SG3249 in comparison to stochastic hD12-SG3249. A 5-day cytotoxicity assay was performed with serial dilutions of test articles (hD12-SSC-SG3249, stochastic hD12-SG3249 and non-targeting control Denosumab-SG3249) starting at 50 nM on lung cancer cell line H1975. The H1975 subcutaneous xenograft model in female Nu/Nu mice was treated with test articles (hD12-SSC-SG3249, hD12-SG3249 and PBS control) at dose concentrations of 0.5 and 0.125 mg/kg *via* single intravenous injection at an average tumor size of 200-250 mm^3^. A rat pharmacology study was performed to assess serum half-life and general tolerability over a 21-day period. **(A)** Potency of hD12-SSC-SG3249 in comparison to stochastically conjugated ADC and non-targeting control ADC in the medium-low cMet lung cancer cell line H1975 in duplicates. Experiment was repeated at least 3 times. A representative experiment is shown. **(B)** Tumor volumes of the subcutaneous H1975 lung cancer xenograft model after intravenous administration of a single dose site-specific hD12-SSC-SG3249, stochastic hD12-SG3249 and 1xPBS vehicle control. Each group contained 8 animals. Statistics: 1-way ANOVA with Dunnett’s multi-comparison (* P<0.05, ** P<0.01, *** P<0.001). **(C)** Time-concentration curves of TR1801-ADC in rats (3 animals per group). 2 different PK ELISA methods (total antibody and intact ADC) were used to measure the serum concentrations and analyze the circulating half-life and deconjugation of TR1801-ADC.**(D)** %bodyweight change in male Sprague-Dawley rats after single intravenous bolus administration of site-specific hD12-SSC-SG3249 at 2, 1.5, 1 and 0.5 mg/kg (3 animals per dose group)

**Suppl. Table S1.**

**TR1801-ADC quality attributes.**

**Suppl. Table S2.**

**Affinity and species cross-reactivity of mouse and humanized cMet P3D12 antibody clone.**

|  |  | **human cMet** | |  | **rat cMet** | |  |
| --- | --- | --- | --- | --- | --- | --- | --- |
|  | **Clone** | **ELISA EC_50_ (μg/mL)** | **SPR kD(nM)** |  | **ELISA EC_50_ (μg/mL)** | **SPR kD(nM)** |  |
|  | P3D12 | 0.05 | 0.88 |  | 0.38 | 15.6 |  |
|  | hD12 | 0.05 | 0.26 |  | 0.33 | 7.0 |  |

**Suppl. Table S3.**

**Cytotoxicity of TR1801-ADC, cMet ADC variants and control ADCs with H1975 and H1373 cancer cell lines.**

Cytotoxicity of TR1801-ADC, cMet ADC variants and control ADCs with H1975 and H1373 cancer cell lines. IC_50_ and % maximal killing were determined in Prism 7 after 5-day incubation and addition of Cell Titer Glo reagent. Experiments were performed in duplicates and repeated at least 1 time.

**Suppl. Table S4.**

**Significance of anti-tumor activity of hD12 PBD drug-linker variants in a H1975 xenograft model.**

Significance of anti-tumor activity of hD12 PBD drug-linker variants in a H1975 xenograft model. One-way ANOVA with Tukey’s multiple comparison was used with Graphpad Prism 7 to compare the different hD12-PBD drug-linker variants to each other at a designated day.

**Suppl. Table S5.**

**Significance of anti-tumor activity of TR1801-ADC at different concentrations and stochastically coupled cMet-vc-MMAE ADC in H1975 and H1373 xenograft models.**

Significance of anti-tumor activity of TR1801-ADC at different concentrations and stochastically coupled cMet-vc-MMAE ADC in H1975 and H1373 xenograft models. One-way ANOVA with Dunnett’s multiple comparison was calculated with Graphpad Prism 7 to compare ADCs to PBS control at a designated day. Significance of TR1801-ADC activity in comparison to cMet-vc-MMAE ADC was calculated using unpaired two-tailed t-tests in Graphpad Prism 7.

**Suppl. Table S6.**

**Significance of anti-tumor activity of TR1801-ADC in PDX models**

Significance of anti-tumor activity of TR1801-ADC at different concentrations and non-targeting ADC in PDX models shown in this manuscript. One-way ANOVA with Dunnett’s multiple comparison was calculated with Graphpad Prism 7 in comparison to PBS control.
